# Supplementary material for: Potential Azo-8-hydroxyquinoline derivatives as multi-target lead candidates for Alzheimer’s disease: An in-depth in silico study of monoamine oxidase and cholinesterase inhibitors
Source: PLoS One. 2025 Jan 30;20(1):e0317261. doi: 10.1371/journal.pone.0317261 (PMC11781659; doi:10.1371/journal.pone.0317261)
Supplement: S1 Table — (DOCX) [file pone.0317261.s001.docx]

# Potential Azo-8-Hydroxyquinoline Derivatives as Multi-Target Lead Candidates for Alzheimer's Disease: An In-Depth In Silico Study of Monoamine Oxidase and Cholinesterase Inhibitors

Fatima Zahra Guerguer ^(1)^, Bouchra Rossafi ^(1)^, Oussama Abchir ^(1)^, Yasir S. Raouf ^(2)^,
Dhabya Bakhit Albalushi ^(2)^, Abdelouahid Samadi ^(2, *)^, and Samir Chtita ^(1, *)^

# Laboratory of Analytical and Molecular Chemistry, Faculty of Sciences Ben M’Sik, Hassan II University of Casablanca, Morocco

# Department of Chemistry, College of Science, United Arab Emirates University, Al Ain P.O. Box 15551, United Arab Emirates

^(*)^ Correspondence: A.S.: [samadi@uaeu.ac.ae](mailto:samadi@uaeu.ac.ae) ; S.C.: [samirchtita@gmail.com](mailto:samirchtita@gmail.com)

**Supplementary material**

**Table S1: 2D structures of the Azo-8HQ molecules studied**

**Group 1:**

| **Molecule** | **Structure** |
| --- | --- |
| **1a** |  |
| **2a** |  |
| **3a** |  |
| **4a** |  |
| **5a** |  |
| **6a** |  |
| **7a** |  |
| **8a** |  |
| **9a** |  |
| **10a** |  |
| **11a** |  |
| **12a** |  |
| **13a** |  |
| **14a** |  |
| **15a** |  |
| **16a** |  |
| **17a** |  |
| **18a** |  |
| **19a** |  |
| **20a** |  |
| **21a** |  |
| **22a** |  |
| **23a** |  |

**Group 2: b) R = H, c) R = CH_3_**

| **Molecule** | **Structure** |
| --- | --- |
| **1b**  **1c** |  |
| **2b**  **2c** |  |
| **3b**  **3c** |  |
| **4b**  **4c** |  |
| **5b**  **5c** |  |
| **6b**  **6c** |  |
| **7b**  **7c** |  |
| **8b**  **8c** |  |
| **9b**  **9c** |  |
| **10b**  **10c** |  |
| **11b**  **11c** |  |
| **12b**  **12c** |  |
| **13b**  **13c** |  |
| **14b**  **14c** |  |
| **15b**  **15c** |  |
| **16b**  **16c** |  |
| **17b**  **17c** |  |
| **18b**  **18c** |  |
| **19b**  **19c** |  |
| **20b**  **20c** |  |
