# Supplementary material for: Potential Azo-8-hydroxyquinoline derivatives as multi-target lead candidates for Alzheimer’s disease: An in-depth in silico study of monoamine oxidase and cholinesterase inhibitors
Source: PLoS One. 2025 Jan 30;20(1):e0317261. doi: 10.1371/journal.pone.0317261 (PMC11781659; doi:10.1371/journal.pone.0317261)
Supplement: Attachment — Submitted filename: comments (1).docx [file pone.0317261.s002.docx]

Dear editor,

Have a nice day. The authors screened computationally 63 Azo-8HQ derivatives against three enzymes acetylcholinesterase (AChE), butyrylcholinesterase (BuChE), and monoamine oxidase B (MAO-B) as promising multi-target candidates for the treatment of AD. Different in *in silico* studies were carried out. This work needs minor modification before its publication as follows.

1- At least the 2D structures of the compounds 14c and 17c should be drawn in the manuscript.
2- The manuscript needs revision form native English speaker.
3- ACh found in postsynaptic neurons and neuromuscular regions, is essential for nerve impulse transmission not AChE
4- What is the basic pharmacophoric features on which the tested compounds were selected?

Best regards
